# Rapid detection of *Galba truncatula* in water sources on pasture-land using loop-mediated isothermal amplification for control of trematode infections

**DOI:** 10.1186/s13071-020-04371-0

**Published:** 2020-09-30

**Authors:** Chelsea N. Davis, Fiona Tyson, David Cutress, Emma Davies, Dewi Llyr Jones, Peter M. Brophy, Alex Prescott, Michael T. Rose, Manod Williams, Hefin Wyn Williams, Rhys Aled Jones

**Affiliations:** 1 Institute of Biological, Environmental and Rural Sciences, Aberystwyth University, Aberystwyth, UK; 2 Coleg Cambria, Llysfasi, Ruthin Road, Ruthin, Denbighshire UK; 3 Tasmanian Institute of Agriculture, University of Tasmania, Sandy Bay, TAS Australia

**Keywords:** Loop-mediated isothermal amplification, Environmental DNA, Farm management, Trematode, Fluke, *Galba truncatula*, *Fasciola hepatica*, *Calicophoron daubneyi*

## Abstract

**Background:**

Fascioliasis caused by the trematodes *Fasciola hepatica* and *F. gigantica*, is a global neglected zoonotic disease estimated to cost the livestock industry over €2.5 billion annually. Farm management measures and sustainable use of anthelmintics can, in principle, effectively control trematode infection in livestock and reduce the rate of developing anthelmintic resistance. Previously, we designed an environmental DNA (eDNA) assay to identify a common trematode intermediate host, the freshwater snail *Galba truncatula*, in water sources to measure specific trematode infection risk areas on pasture-land. To improve this procedure, we now report a loop-mediated isothermal amplification (LAMP) assay to identify *G. truncatula* eDNA.

**Methods:**

A LAMP assay was designed and optimised (e.g. temperature, time duration and primer concentration) to identify *G. truncatula* DNA. The ability of the LAMP assay to target *G. truncatula* DNA was identified, and LAMP assay limit of detection was investigated in comparison to conventional PCR. In the field, 48 water samples were collected from stream, ditch and water pool habitats in four locations at two Aberystwyth University farms over a seven week period to investigate the applicability of the LAMP assay for use on eDNA samples, in comparison to conventional PCR.

**Results:**

The LAMP assay delivered detectable results in 30 min at 63 °C. The assay discriminated between *G. truncatula* DNA and non-target DNA, presenting a level of DNA detection comparable to conventional PCR. No significant difference was found between the ability of the LAMP and PCR assay to identify *G. truncatula* eDNA in water samples. Kappa coefficient analysis revealed a moderate level of agreement between LAMP and PCR assays.

**Conclusions:**

This study demonstrated that the LAMP assay can detect *G. truncatula* eDNA in a simple and rapid manner. The LAMP assay may become a valuable tool to determine optimum pasture management for trematode parasite control.
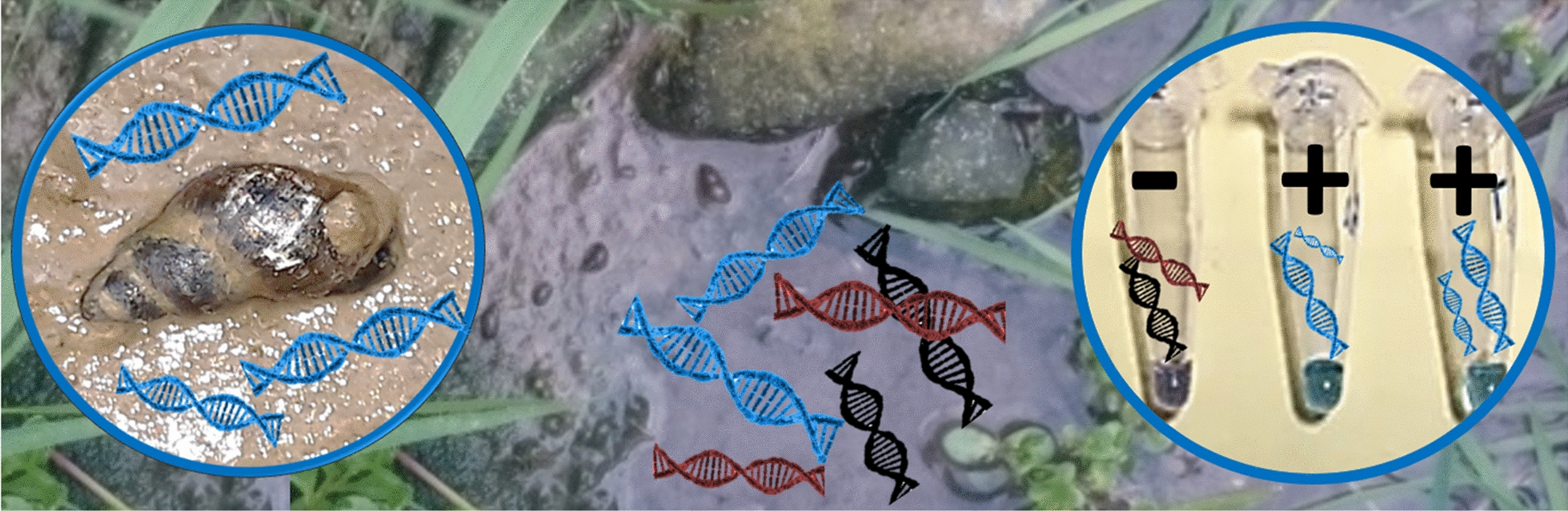

## Background

*Galba truncatula* is an intermediate host for the trematodes *Fasciola hepatica* and *Calicophoron daubneyi* [1]. Trematode parasite stages develop and multiply *via* polyembryony in *G. truncatula* to produce cercaria. These encyst on herbage as infective metacercaria, which consequently infect definitive hosts, such as ruminant species [2, 3]. *Fasciola hepatica* is a trematode of worldwide economic importance [4] as the disease it causes is estimated to cost the global livestock production industry €2.5 billion annually [5]. Furthermore, at least 2.4 million people are currently infected in over seventy countries, with millions more at risk of this food-borne disease [4]. In addition, *C. daubneyi* is an emerging trematode of concern in temperate countries [6–8]. In the absence of vaccines, trematode control in the definitive host is primarily *via* anthelmintic administration; however, anthelmintic resistance and the limited availability of novel anthelmintic compounds threatens sustainable control [9].

Farm management combined with the effective use of anthelmintics has been found to control the disease and prevent resistance [10, 11]. Farm management practices that can reduce host parasite exposure include improving grazing management, draining pasture, fencing off wet pastures, using water troughs, and managing animal stocking rate [12–14]. Currently, regional liver fluke infection risk may be remotely forecasted using climatic models such as the Ollerenshaw index [15], whilst research is ongoing to develop geographical information system and satellite imagery analysis tools that aim to identify specific infection risk areas in fields [16–18]. However, even in areas where climatic and environmental conditions seem suitable, intermediate snail hosts may be absent leading to zero trematode infection risk for livestock grazing those areas [12, 19].

Recently, a water-based environmental DNA (eDNA) assay has been designed to identify the presence of *G. truncatula* in water sources on pasture-land [20]. An optimised *G. truncatula* eDNA assay would be cost-effective, simple and rapid to undertake, meaning this method would be ideal to implement localised and seasonal farm management practices on a per field basis. Loop-mediated isothermal amplification (LAMP) assays have been used to detect trematode species in field and laboratory settings [21]. LAMP assays are highly sensitive and easy to undertake, requiring only designed primers, DNA polymerase and a water bath or heat block to complete the reaction, and the results can be analysed visually [22–27]. LAMP assays are also highly specific as they exploit up to six primers for target amplification and are therefore less prone to non-target DNA issues than conventional PCR tests [22, 28, 29]. Consequently, a LAMP assay designed to detect the *G. truncatula* DNA in combination with a previously designed water-based eDNA assay, could be a valuable new tool to identify farm trematode infection risk areas on pasture-land.

## Methods

### LAMP design

A set of six LAMP PCR primers (Sigma-Aldrich, St. Louis, USA); a forward inner primer (FIP), a backward inner primer (BIP), two outer primers (F3 and B3) and two loop primers (LoopB and LoopF) were designed with assistance by Mast Group Ltd (Bootle, UK) to amplify *G. truncatula* DNA according to published *G. truncatula* internal transcribed spacer 2 (ITS2) sequences (GenBank: AJ296271.1, KT280448.1. KT781252.1, MH561919.1, JX536270.1; Table 1). The primers were aligned and checked for specificity to *G. truncatula* DNA using comparative genome Basic Local Alignment Search Tool (BLAST) analysis.

**Table 1:** Primers targeting *G. truncatula* ITS2 DNA sequence used for LAMP assay

Primers	Sequence (5′-3′)	Amplicon size (bp)
F3	CTCGGCGATGGTTGGATA	18
B3	ATCTCGTCCGATCTGAGG	18
FIP	CCGAGAACGCCACGATAATTGTCCGTTCATCTCGTAAC	28
BIP	AGTCCATGGCATCGCAGCACCACGTAGCGTCTTAGA	36
LoopF	CTGCCTGGCGGTAGAGAA	18
LoopB	GTGGGTGGAGAACAAGGG	18

The final reaction mixture (10 μl) contained 3  pmol of outer primer (F3 and B3), 25 pmol of inner primer (FIP and BIP) and 12 pmol of loop primer (loop B and loop F). MAST ISOPLEX® DNA *Lyo* kit (Mast Group Ltd) and 120 µM hydroxy naphthol blue (HNB) were used to make the mastermix, before adding 0.8 μl of primer mix and 1 μl of DNA. Amplification was conducted at 63 °C for 30 min in a thermocycler (TC-4000, Techne, Stone, UK).

The LAMP reaction conditions were optimised with different parameters including assay temperature (60.4–64.7 °C), primer concentration (between 22–28 pmol for FIP and BIP; 10–14 pmol loop F and loop B; and 2.5–3.5 pmol F3 and B3) and incubation time (10–40 min) using triplicate biological replicates. Each LAMP assay run included a negative control (DNAase-free water only) and a positive control (genomic DNA extracted from *G. truncatula* snails). Samples were considered positive when the LAMP product showed a colour change from the violet-coloured negative control, to a sky-blue colour similar to the positive control, in addition to the presence or absence of banding after gel electrophoresis of LAMP product.

LAMP primer cross-reactivity was investigated by undertaking LAMP reactions using triplicate biological replicates of DNA extractions from the lymnaeid snails *Radix balthica*, *Lymnaea fuscus*, *Omphiscola glabra*, *Stagnicola palustris* and *Lymnaea stagnalis*. *Lymnaea fuscus* specimens were gifted by the National Museum Wales, *O. glabra* DNA extracts were gifted by the Royal Zoological Society of Scotland (RZSS) and *L. stagnalis* specimens were gifted by Aberystwyth University. Specimens of *R. balthica* and *S. palustris* were collected in the field after identification with a gastropod dichotomous key [30].

The lower limit of detection of the LAMP assay was determined by 10-fold dilutions of a known concentration of *G. truncatula* genomic DNA extract. The last dilution where all three replicates were recorded positive was considered as the detection limit.

### DNA extraction and conventional PCR

Genomic DNA from all snail species except *O. glabra* was extracted using an adaptation of a Chelex® (Bio-Rad, Hercules, USA) method [31], as previously described [32]. *Omphiscola. glabra* DNA extraction was undertaken at RSZZ using a blood and tissue kit (Qiagen, Hilden, Germany). Extracted DNA samples were stored at – 20 °C until use.

LAMP assay performance was compared against a conventional PCR method previously described [20]. Modifications to the original method included the total reaction volume, where a 10 μl master mix was created containing 5 μl of Platinum™ Green Hot Start PCR Master Mix (Thermo Fisher Scientific, Hayward, USA), 0.5 μl of each 10 μM primer, 1 μl of the extracted DNA and nuclease-free water. A non-template control (DNAase-free water) as a negative control and a positive control (genomic DNA extracted from *G. truncatula* snails) were included for each PCR run. The LAMP and PCR products (10 μl) were analysed by 2% agarose gel (agarose: Bioline, Tris Acetate-EDTA buffer: National Diagnostics) electrophoresis (Midi Plus Horizontal Gel System, Fisherbrand, Hampton, USA) stained with SYBR safe DNA gel stain (Invitrogen, Carlsbad, USA) and observed under UV transillumination (Genoplex, VWR, Radnor, USA) or Typhoon FLA 9000 Gel Imaging Scanner (GE Healthcare, Chicago, USA).

The lower limit of detection of the conventional PCR was determined by 10-fold dilutions of a known concentration of *G. truncatula* genomic DNA extract. The last dilution with all triplicate replicates testing positive was considered as the detection limit.

### eDNA collection and extraction

**Fig. 1:**
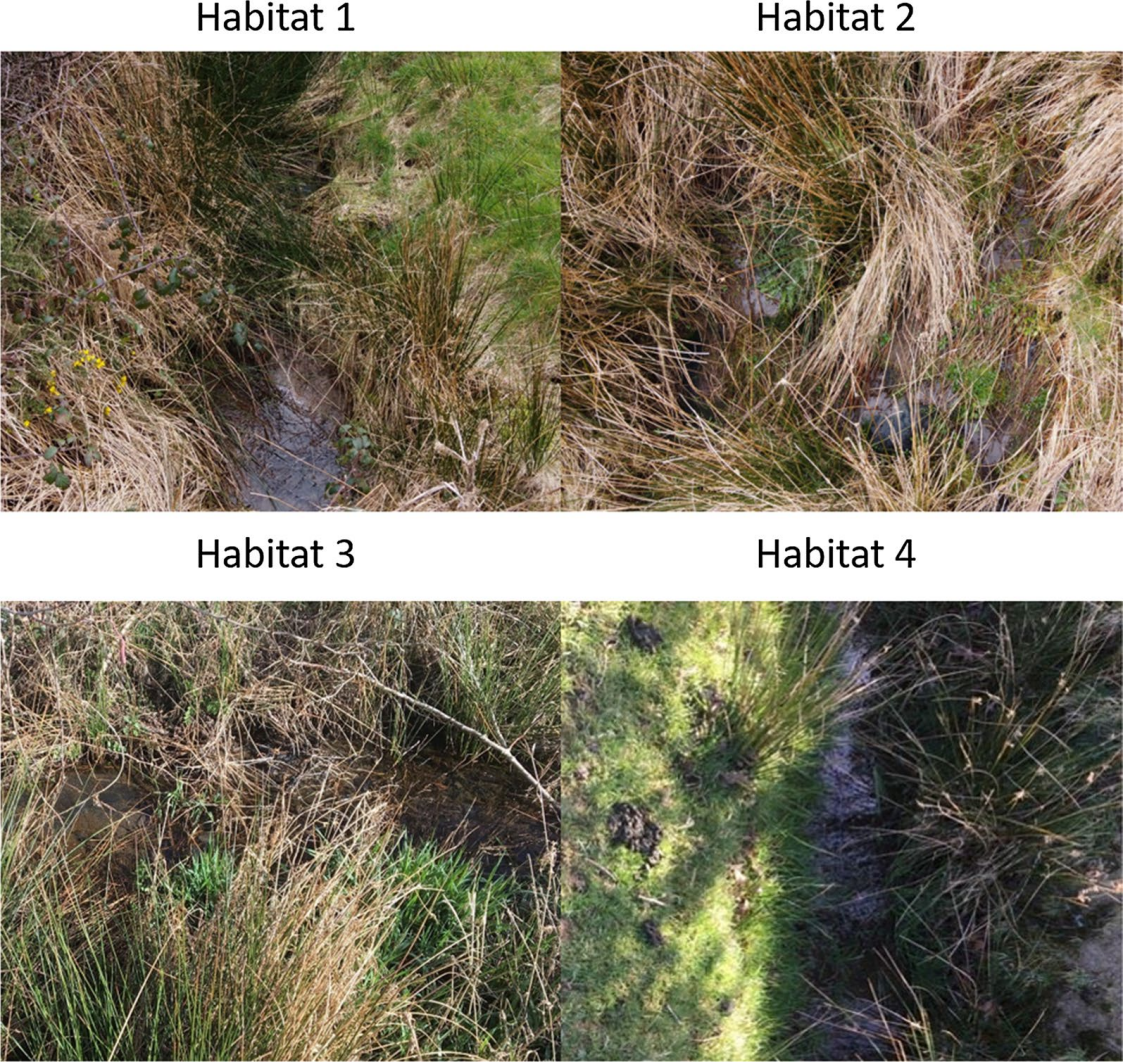
Photographic images of four habitats where eDNA samples were collected. These four locations were identified to be potentially suitable for *G. truncatula* habitation at two Aberystwyth University farms were: Habitat 1, slow moving stream; Habitat 2, standing water pools; Habitat 3, slow moving stream; and Habitat 4, drainage ditch

To investigate the LAMP assay’s capabilities of identifying the presence of *G. truncatula* on pasture-land, water samples were collected and analysed from four locations at Farm A (52.424226N, − 4.051630W) and Farm B (52.425258N, − 4.029358W) which are both located at Aberystwyth University, Ceredigion, Wales, UK, over a seven-week period (3rd March-13th June 2019). In each sampling location, two replicate samples were collected at six time points in this period, giving a total of 48 samples. Each sampling location were in *G. truncatula* habitats enrolled in a long-term eDNA research project. Sampling location types included two slow moving streams, a boggy area containing standing water pools and a drainage ditch (Fig. 1).

A filter-based eDNA capture protocol was used throughout this study following the methodology of Jones et al. [20]. A blank control (500 ml distilled water) was undertaken during every day of eDNA collection. Filters were stored at − 20 °C until use. Non-disposable equipment were soaked in 7% sodium hypochlorite overnight, before being rinsed in water and dried to avoid cross-contamination between sample collections [33].

DNA was extracted from filter samples using the DNeasy® PowerSoil® kit (Qiagen). Each filter, including blank controls, was homogenised using a sterile pipette tip and the whole filter was then subjected to DNA extraction *via* the PowerSoil® kit protocol. Each eDNA sample was tested in triplicate by both LAMP and PCR assays, where the sample was deemed positive if one or more of the triplicate technical replicates gave a positive result.

### Statistical analysis

The success of the newly developed LAMP assay in detecting *G. truncatula* DNA in extracted eDNA samples compared to PCR was analysed statistically using a generalized logistic regression mixed model in SPSS (v.25). The subject variable was each eDNA sample, with DNA amplification method (LAMP or PCR) the within subject variable. The dependent variable was the outcome of each DNA amplification test (positive or negative). The DNA amplification method (LAMP or PCR) was inserted as a fixed factor in the model to identify if any differences in DNA amplification from each sample was seen between both methods. The habitat and sampling time-point were inserted into the model as random factors to identify if any differences in test performance was influenced by sampling location and period. Outcomes were deemed significant if *P* < 0.05. Kappa coefficient analysis with 95% confidence level was undertaken to assess the agreement between LAMP and PCR assays in amplifying eDNA using SPSS (v.25).

## Results

### Comparison of LAMP and conventional PCR limit of detection

**Fig. 2:**
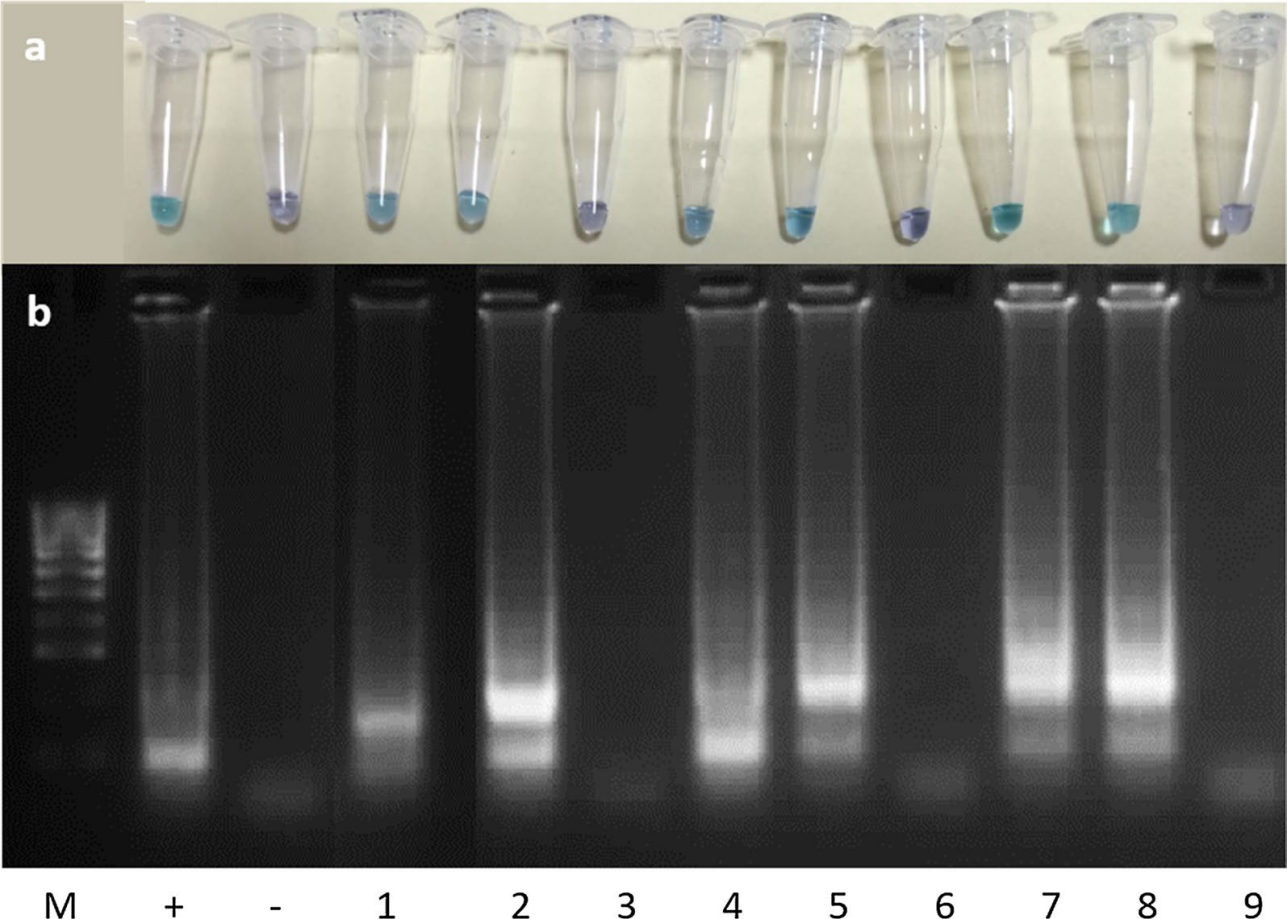
Lower limit detection and optimum primer concentration of *G. truncatula* LAMP assay. This was determined by undertaking (**a**) LAMP assay and (**b**) confirmation of results of the LAMP assay products using agarose gel (2%) electrophoresis. Lane M: 100 bp DNA Ladder (Thermo Fisher Scientific). Lane + (positive control of 34.9 ng/μl *G. tuncatula* genomic DNA extract) and Lane− (negative control) were both undertaken at primer concentration 3 pmol of F3 and B3 primer, 25 pmol of FIP and BIP primer and 12 pmol loop B and loop F primer. Lane 1 (10^4^ dilution of *G. tuncatula* genomic DNA extract), Lane 2 (10^5^ dilution of *G. tuncatula* genomic DNA extract) and Lane 3 (10^6^ dilution of *G. tuncatula* genomic DNA extract) were undertaken at primer concentration 2.5 pmol of F3 and B3 primer, 22 pmol of FIP and BIP primer and 10 pmol of loop B and loop F primer. Lane 4 (10^4^ dilution of *G. tuncatula* genomic DNA extract), Lane 5 (10^5^ dilution of *G. tuncatula* genomic DNA extract) and Lane 6 (10^6^ dilution of *G. tuncatula* genomic DNA extract) were undertaken at primer concentration 3 pmol of F3 and B3 primer, 25 pmol of FIP and BIP primer and 12 pmol of loop B and loop F primer. Lane 7 (10^4^ dilution of *G. tuncatula* genomic DNA extract), Lane 8 (10^5^ dilution of *G. tuncatula* genomic DNA extract) and Lane 9 (10^6^ dilution of *G. tuncatula* genomic DNA extract) were undertaken at primer concentration 3.5 pmol of F3 and B3 primer, 28 pmol of FIP and BIP primer and 14 pmol of loop B and loop F primer

To determine the limit of detection of the LAMP assay, 10-fold dilutions (10^1^–10^9^) of a known concentration of *G. truncatula* genomic DNA extract and a range of primer concentrations were used for LAMP experiments. The lower limit of detection for the LAMP assay was found to be 10^5^-fold dilution (0.349 pg/μl) when using 22–28 pmol FIP and BIP primer, 10–14 pmol loop F and loop B primer and 2.5–3.5 pmol F3 and B3 primer in the reaction (Fig. 2).

**Fig. 3:**
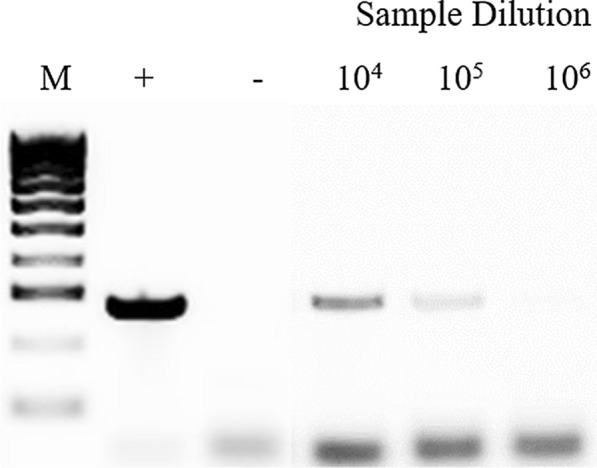
Lower limit of detection for *G. truncatula* conventional PCR. This was determined by making 10-fold dilutions ranging from 10^4^–10^6^ of 50 ng/μl *G. truncatula* genomic DNA extract samples. Lane M: 100 bp DNA Ladder (Thermo Fisher Scientific); Lane +: positive control; Lane −: no-template negative control

Similar 10-fold dilutions were used to compare the limit of detection of the LAMP assay with conventional PCR. PCR was identified as having a similar lower limit detection (10^5^-fold dilution, e.g. 0.5 pg/μl) compared to the LAMP assay in these conditions (Fig. 3).

### LAMP temperature optimisation

**Fig. 4:**
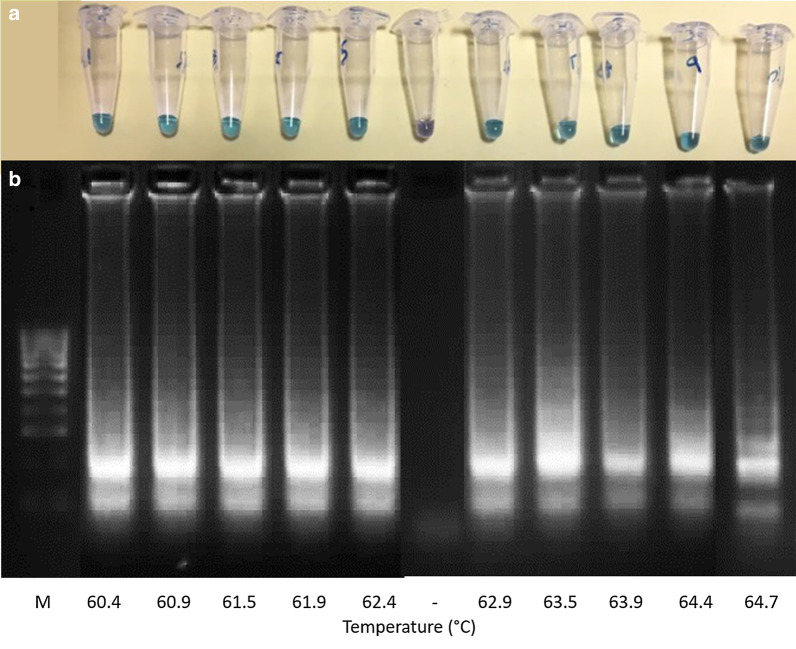
Temperature optimisation of *G. truncatula* LAMP assay. This was determined by undertaking a LAMP assay (**a**) and confirmation of results of the LAMP assay products using agarose gel (2%) electrophoresis (**b**). Temperatures ranged between 60.4–64.7 °C of 34.9 ng/μl *G. truncatula* genomic DNA extract sample concentration. Lane M: 100 bp DNA Ladder (Thermo Fisher Scientific); Lane –: no-template negative control at 63 °C

All reaction temperatures (60.4–64.7 °C) investigated amplified *G. truncatula* genomic DNA during temperature optimisation of the LAMP assay (Fig. 4). As the MAST ISOPLEX® DNA *Lyo* kit recommends undertaking LAMP assays at 63 °C, this temperature was chosen for further experimentation.

### LAMP reaction duration optimisation

**Fig. 5:**
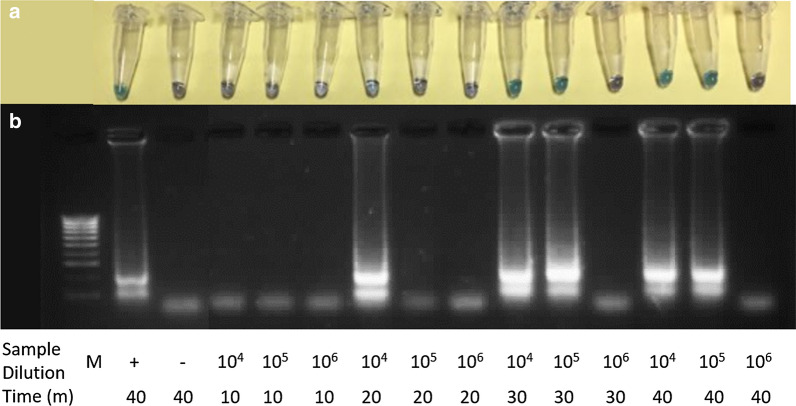
Optimum time duration of *G. truncatula* LAMP assay. This was determined by undertaking a LAMP assay (**a**) and confirmation of results of the LAMP assay products using agarose gel (2%) electrophoresis (**b**). Time durations ranged from 10–40 min and 10-fold dilutions ranged from 10^4^–10^6^ of 34.9 ng/μl *G. truncatula* genomic DNA extract sample concentration. Lane M: 100 bp DNA Ladder (Thermo Fisher Scientific); Lane +: *G. truncatula* positive control; Lane−: no-template negative control

To determine the time duration in which the LAMP assay identified a positive outcome, 10-fold dilutions (10^4^–10^6^) of *G. truncatula* genomic DNA extract were subjected to the LAMP assay for four time durations (10, 20, 30 and 40 min) (Fig 5). The LAMP assay showed a positive result in 20 min at 10^4^ fold dilution of *G. truncatula* genomic DNA extract (e.g. 3.49 pg/μl), although this time duration reduced the limit of detection of the LAMP assay by 10-fold. A time duration of 30 min resulted in a positive outcome at the lower limit of detection for the LAMP assay (10^5^-fold dilution = 0.349 pg/μl), so was chosen as the optimum LAMP assay time duration.

### LAMP primer cross-reactivity

**Fig. 6:**
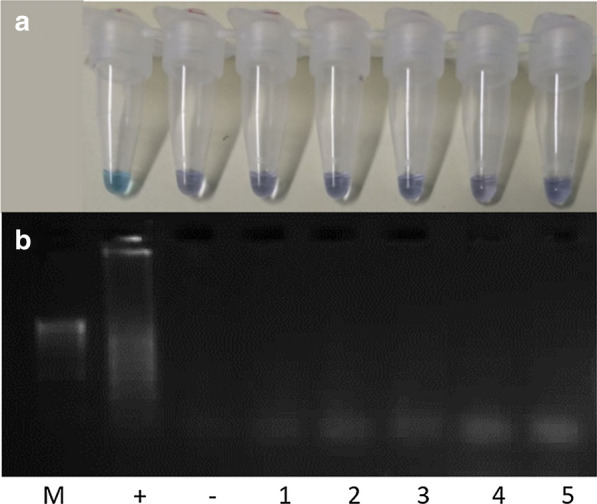
Cross-reactivity of *G. truncatula* LAMP assay. This was determined by undertaking a LAMP assay (**a**) and confirmation of results of the LAMP assay products using agarose gel (2%) electrophoresis (**b**). In panels **a** and **b**: M, 100 bp DNA Ladder (Thermo Fisher Scientific); +, *G. truncatula* positive control; −, no-template negative control; 1, *S. palustris* (140.6 ng/μl); 2, *L. stagnalis* (143.3 ng/μl); 3, *R. balthica* (167.2 ng/μl); 4, *L. fuscus* (218.2 ng/μl); 5, *O. glabra* (164.9 ng/μl)

Cross reactivity of the designed LAMP primers was investigated by undertaking the LAMP assay with genomic DNA of five snail species belonging to the family Lymnaeidae: *S. palustris*, *O. glabra*, *L. stagnalis*, *L. fuscus* and *R. balthica* which are closely related to and live in similar environments to *G. truncatula*. None of these snail species showed a positive result when the LAMP assay was undertaken, demonstrating that the designed LAMP primers showed the ability to discriminate between *G. truncatula* DNA and non-target snail DNA (Fig. 6).

### Detection of *G. truncatula* eDNA in water sources using the LAMP assay

Water samples from pasture fields were used to test the applicability of the LAMP assay to identify *G. truncatula* DNA in the environment in comparison to PCR (Table 2). Both assays reported similar results, where samples taken from Farm A showed 17/24 and 16/24 positive results for *G. truncatula* DNA using the LAMP and PCR assay, respectively. At Farm B, 10/24 and 11/24 positive results for *G. truncatula* DNA were found using the LAMP and PCR assay, respectively.

**Table 2:** Comparison of *G. truncatula* LAMP and PCR assays using environmental water samples

Date collected	Habitat	LAMP (*n*/*N*)	PCR (*n*/*N*)
Farm A			
03/05/2019	1	2/2	0/2
03/05/2019	2	0/2	0/2
09/05/2019	1	1/2	1/2
09/05/2019	2	1/2	1/2
16/05/2019	1	1/2	1/2
16/05/2019	2	2/2	2/2
30/05/2019	1	2/2	2/2
30/05/2019	2	2/2	2/2
06/06/2019	1	2/2	2/2
06/06/2019	2	1/2	1/2
13/06/2019	1	2/2	2/2
13/06/2019	2	1/2	2/2
Farm B			
03/05/2019	3	1/2	0/2
03/05/2019	4	0/2	0/2
09/05/2019	3	0/2	1/2
09/05/2019	4	0/2	1/2
16/05/2019	3	1/2	2/2
16/05/2019	4	1/2	1/2
30/05/2019	3	1/2	1/2
30/05/2019	4	2/2	1/2
06/06/2019	3	1/2	0/2
06/06/2019	4	0/2	1/2
13/06/2019	3	2/2	2/2
13/06/2019	4	1/2	1/2

*Notes*: eDNA samples were collected in four locations at Farm A and Farm B over a 7-week period. Area characteristics of the habitats were: 1, stream; 2, water pools; 3, stream; 4, ditch (as shown in Fig. 1)

*Abbreviation*: n/N, positive/examined

There was no significant difference between the ability of the LAMP and PCR assays to identify the presence of *G. truncatula* eDNA in environmental samples (*P* = 1.000) (Table 3) with no significant random effect of habitat (*P* = 0.409) and sampling time (*P* = 0.222) on the results. Kappa coefficient analysis (Table 4) revealed a moderate level of agreement (Kappa = 0.577) between assays [34].

**Table 3:** Generalized logistic regression mixed model comparing eDNA *G. truncatula* LAMP and PCR assays

Variable	β	SE	*P*-value
Intercept	− 0.296	0.815	0.717
LAMP assay	0.000	0.444	1.000

Random effects	Variance		
Intercept	4.074		
Habitat	0.426	0.516	0.409
Time	1.155	0.945	0.222

*Abbreviation*: SE, standard error

**Table 4:** Kappa agreement comparing eDNA *G. truncatula* LAMP and PCR assays

No. of samples				
PCR results	LAMP positive	LAMP negative	Total	Kappa value
Positive	16	5	21	0.577
Negative	5	22	27	
Total	21	27	48	

*Notes*: The Kappa coefficient was undertaken with 95% confidence level and conducted using SPSS (v.25). The standard error = 0.119 and *P* < 0.001

## Discussion

The success of host trematode parasite transmission is heavily influenced by *G. truncatula* population dynamics in the environment [3]. Current methods for identifying precise trematode infection risk areas in fields by physically detecting *G. truncatula* presence are labour-intensive, require expertise, and are time-consuming and costly [12, 19]. In this paper, a practical LAMP assay has been designed to detect the eDNA of trematode intermediate host, *G. truncatula,* in water samples. Detection of *G. truncatula* eDNA may indicate trematode infection risk areas on pasture which will allow farm management practices to be applied to reduce trematode infection risk in livestock grazing specific pastures.

The limit of detection of the LAMP assay (0.349 pg/μl) was comparable to conventional PCR (0.5 pg/μl), when using *G. truncatula* DNA extract in the reaction. Similar results were found developing a LAMP assay to detect *O. viverrini* in human faeces where the limit of detection for the reaction was between 1 pg and 100 fg of template, although the LAMP assay limit of detection was 100-fold greater, compared to conventional PCR [35]. In agreement with the present study, LAMP assays designed to detect *Fasciola* spp. in the faeces of sheep, *O. viverrini* in human faecal stools and *Amphimerus* spp. in human faeces showed the same 10-fold limit of detection as conventional PCR [36–38]. In contrast, investigations have found different outcomes upon LAMP assay DNA detection limits and adverse results when LAMP assays are compared to conventional PCR [39–41]. These inconsistent assay outcomes are likely due to the quality of the LAMP assay being dependent upon the regions that the primers were designed [22]. In this study, the ITS2 gene was chosen as the target gene for LAMP assay design, because the ITS2 sequence is known to occur in tandem repeats in the ribosomal DNA; it is therefore likely to be present in larger quantities in the environment [42, 43].

It is recognised that qPCR assays have lower detection limits than PCR assays [44]. Optimisation of a qPCR assay upon the detection of *F. hepatica* and *A. tomentosa* in water samples determined a limit of detection of 14 fg or 50 fg, respectively, where the DNA detection limit was 100-fold greater than conventional PCR [45]. Compared to LAMP assay, qPCR is less practical to undertake due to the requirement of a precise, expensive instrument for amplification and a skilful labourer needed for detection of the amplified products [22].

All the reaction temperatures (60.4–64.7 °C) investigated amplified *G. truncatula* genomic DNA when optimising the LAMP assay, so 63 °C was chosen for further experimentation as recommended by the LAMP kit used. LAMP assays are most efficient at these temperatures because they allow best performance of *Bst* DNA polymerase and double-stranded DNAs are at dynamic equilibrium around this temperature, so one of the LAMP primers (FIP) can anneal to the DNAs without a denaturing step to initiate synthesis [22, 24]. The fact that the LAMP assay reaction can be undertaken under isothermal conditions using inexpensive equipment, means that detection of trematode infection risk areas on pasture-land can be conducted in less advanced field laboratories without the expense of thermocyclers and electrophoresis instrumentation.

LAMP assay results can be visualised in 30 min to detect *G. truncatula* genomic DNA extract at the limit of detection. Comparatively, the conventional PCR reaction took a further two hours to complete than the LAMP assay, and required gel electrophoresis to determine an outcome. A LAMP assay designed to detect *F. hepatica* and *F. gigantica* using four species-specific primers, took 45 min to obtain amplification and observe a visual result [26]. The addition of loop primers accelerates the LAMP reaction, as they hybridize to the stem loops, except for the loops that are hybridized by the inner primer, priming strand replacement DNA synthesis [28]. Using six primers, a LAMP assay designed to detect *Schistosoma mansoni* obtained positive results in 20 min, 30 min and 50 min when using 1 ng, 1 pg or 1 fg DNA template, respectively [46]. The ability of a LAMP assay reaction to visualise an immediate result in 30 min, presents a practical tool to quickly and effectively make decisions upon management of pasture-land. However, advancements are needed to develop a rapid field-based eDNA extraction method to fully capitalise on the capabilities of LAMP assay.

The LAMP primers showed the ability to discriminate between *G. truncatula* DNA and non-target DNA, as five closely related snails that live in similar environments to *G. truncatula* tested negative using the LAMP assay. Amplification of non-target DNA present in the sample is unlikely in LAMP assays due to at least six distinct regions on the DNA target being recognised by at least four primers in the reaction [22, 28]. This suggests that the LAMP assay is robust enough to detect *G. truncatula* eDNA in areas where other snails co-occur.

The LAMP assay identified the presence of *G. truncatula* DNA in environmental samples (e.g. Farm A: 17/24, Farm B: 10/24 positive results) with no significant difference to conventional PCR *G. truncatula* eDNA identification (e.g. Farm A: 16/24, Farm B: 11/24 positive results). Even though both farms showed similar positive results using both assays, three samples at Farm A and seven samples from Farm B showed conflicting results at different sampling points and habitat locations. This explains why the Kappa coefficient analysis showed a moderate level of agreement between LAMP and PCR assays. Similarities between PCR and LAMP assay results have also been reported detecting schistosome-infected snails, whereby 3/90 *Biomphalaria pfeifferi* were found infected with *S. mansoni* using both PCR and LAMP assays in field habitat locations, and 54/103 and 50/103 *Bulinus* spp. snails were found infected with *S. mansoni* using both PCR and LAMP assays, respectively [47]. In contrast, conventional PCR underestimated the presence of *C. sinensis* in snail, fish and shrimp species collected in 11/106 samples compared to LAMP, where the Kappa statistic showed the techniques had high consistency [48].

Environmental samples are known to be rich in PCR inhibitors (e.g. humic acids, potassium dichromate, formaldehyde and phenols) which can interfere with the PCR amplification [49]. Inhibition of PCR reactions by substances present in environmental samples is particularly important in low concentration samples and surface water samples [50]. LAMP assays have been found to be less affected by non-target DNA in comparison to PCR [22, 29]. This is due to the *Bst* DNA polymerase used in LAMP amplification having activity at high temperatures which reduces non-specific priming and that *Bst* DNA polymerase is more resistant to inhibitors compared to other DNA polymerases [51]. In the present study, inhibitors present in the environmental water samples may have adversely affected the LAMP and PCR assay efficiencies, explaining the Kappa coefficient analysis outcome. Of note, the HNB dye used to visualise the present assay, does not interfere with DNA synthesis by *Bst* DNA polymerase [25]. One of the disadvantages of LAMP is the impracticality of including an internal positive control within each reaction [52]. However, it may be feasible to analyse spiked samples to identify if negative LAMP assays were inhibited [53]. Additionally, it is suggested that further research be conducted to understand to what extent PCR inhibitors associated with confounding factors (e.g. water turbidity and soil type) may affect this LAMP assay.

Because LAMP assays are less affected by non-target DNA, some studies have omitted DNA extraction steps, prior to conducting LAMP reactions [29, 54]. Rapidly heating urine samples in comparison to using urine DNA extraction mini kit or sodium hydroxide/sodium dodecyl sulfate extraction method, provided best results for extracting *Schistosoma haematobium* DNA detectable by the LAMP assay, where the limit of detection was 10-fold higher than when using the commercial kit [55]. Heating samples prior to undertaking LAMP assays has also been applied to blood samples to detect malaria [51, 56] and swaps to diagnose leishmaniasis [57] and trichomoniasis [58]. An eDNA LAMP assay which does not require complex DNA extraction steps, would enhance its value to be used as a routine farm management tool. Therefore, further research should investigate alternative methodology to concentrate water eDNA samples and extract DNA, to improve the practicality of the current LAMP assay.

The LAMP assay developed in this study detects the presence of the trematode intermediate snail host *G. truncatula* in the environment. The eDNA capture and extraction protocol utilised in this study has previously demonstrated a capability of identifying *F. hepatica* and *C. daubneyi* eDNA when used in conjunction with PCR [20]. LAMP assays have been developed to amplify *F. hepatica* DNA [26] and could be incorporated into this protocol to identify the presence of *F. hepatica* in the grazing environment. However, as the eDNA of infective and non-infective *F. hepatica* stages cannot be differentiated, interpreting *F. hepatica* eDNA detection in relation to infection risk would be challenging [20].

To further enhance the practicability of the LAMP assay as a farm management tool, a simple formulated ready-to-use LAMP kit should be made available [59]. A high throughput portable LAMP detection system has already been achieved for the diagnosis of malaria parasites, where DNA extraction to result visualisation can be undertaken within two hours without the need of pipetting and centrifugation [60]. Also, a point-of-care pathogen screening tool has been designed where sample acquisition, preparation, amplification and detection can be undertaken in one disposable tube in a field environment [61]. In the future, the current LAMP assay should be developed to provide real time results for multiple field locations on a farm, so farm management decisions can be promptly made to prevent livestock grazing trematode risk areas.

## Conclusions

This LAMP assay has the ability to detect *G. truncatula* DNA from water sources in the environment. Due to the short reaction time and ability to visualise results immediately using inexpensive equipment, we predict this LAMP assay will be a valuable new tool to rapidly and effectively make decisions to support farm management practices in trematode risk areas, specifically in individual field locations.

## Data Availability

All data generated or analysed during this study are included in this published article.
